# Munc18a Does Not Alter Fusion Rates Mediated by Neuronal SNAREs, Synaptotagmin, and Complexin*

**DOI:** 10.1074/jbc.M114.630772

**Published:** 2015-02-25

**Authors:** Yunxiang Zhang, Jiajie Diao, Karen N. Colbert, Ying Lai, Richard A. Pfuetzner, Mark S. Padolina, Sandro Vivona, Susanne Ressl, Daniel J. Cipriano, Ucheor B. Choi, Niket Shah, William I. Weis, Axel T. Brunger

**Affiliations:** From the Departments of ‡ Molecular and Cellular Physiology,; § Neurology and Neurological Sciences,; ¶ Structural Biology, and; ‖ Photon Science and; the ** Howard Hughes Medical Institute, Stanford University, Stanford, California 94305

**Keywords:** Exocytosis, Membrane Fusion, Neurotransmitter Release, SNARE Proteins, Synaptotagmin, Munc18, Single Vesicle Assay, Complexin, Fusion Machinery, Synaptic Vesicle

## Abstract

Sec1/Munc18 (SM) proteins are essential for membrane trafficking, but their molecular mechanism remains unclear. Using a single vesicle-vesicle content-mixing assay with reconstituted neuronal SNAREs, synaptotagmin-1, and complexin-1, we show that the neuronal SM protein Munc18a/nSec1 has no effect on the intrinsic kinetics of both spontaneous fusion and Ca^2+^-triggered fusion between vesicles that mimic synaptic vesicles and the plasma membrane. However, wild type Munc18a reduced vesicle association ∼50% when the vesicles bearing the t-SNAREs syntaxin-1A and SNAP-25 were preincubated with Munc18 for 30 min. Single molecule experiments with labeled SNAP-25 indicate that the reduction of vesicle association is a consequence of sequestration of syntaxin-1A by Munc18a and subsequent release of SNAP-25 (*i.e.* Munc18a captures syntaxin-1A via its high affinity interaction). Moreover, a phosphorylation mimic mutant of Munc18a with reduced affinity to syntaxin-1A results in less reduction of vesicle association. In summary, Munc18a does not directly affect fusion, although it has an effect on the t-SNARE complex, depending on the presence of other factors and experimental conditions. Our results suggest that Munc18a primarily acts at the prefusion stage.

## Teaser

**Background:** Munc18-1 is an important factor for synaptic transmitter release, but its molecular mechanism remains an enigma.

**Results:** Munc18a does not affect fusion kinetics. It can sequester syntaxin-1A molecules from the syntaxin-1A·SNAP-25 t-SNARE complex.

**Conclusion:** Munc18a has no effect on complete fusion in conjunction with synaptotagmin-1, complexin-1, and neuronal SNAREs.

**Significance:** This work provides new insights into Munc18-1 and its interactions with other synaptic proteins.

## Introduction

Synaptic neurotransmission involves the release of neurotransmitter into the synaptic cleft upon an action potential. The release of neurotransmitters into the synaptic cleft is mediated by the fusion of neurotransmitter-laden synaptic vesicles with the plasma membrane in the active zone upon an increase of the local Ca^2+^ concentration following depolarization. There are several synaptic proteins that have key roles in the Ca^2+^-triggered fusion of synaptic vesicles with the plasma membrane: the neuronal soluble *N*-ethylmaleimide-sensitive factor (NSF)^7^ attachment protein receptors (SNAREs) synaptobrevin-2/VAMP2 (vesicle-associated membrane protein), syntaxin-1A, and SNAP-25 (synaptosome-associated protein 25); synaptotagmin; complexin; and nSec1/Munc-18a (1–3). Whereas SNARE proteins can provide the minimal energy to drive membrane fusion (4) and synaptotagmin-1 is the Ca^2+^ sensor for fast synchronous release (5), the molecular roles of complexin and Munc18a are less certain (6–9). Here we focus on the mechanism of action of Munc18a.

There is intensive debate as to whether Sec1/Munc18 (SM) proteins, which are required components of all membrane trafficking pathways, play a role in facilitating or regulating SNARE complex assembly or if they are involved in the fusion process itself (10, 11). Evidence that the neuronal SM protein Munc18a is involved in more than “just” facilitation or regulation comes primarily from Munc18a knock-out studies in mice (12) that resulted in complete loss of neurotransmitter release. However, Munc18a knockout also resulted in a 70% reduction in total syntaxin-1 protein level in brain lysates of the knock-out mice and reduced syntaxin-1 mRNA levels (13). Nonetheless, the residual syntaxin-1 was correctly targeted to the synapse and formed SDS-resistant SNARE complex (14), so the reduction in syntaxin-1 levels in the Munc18a knock-out mice may not explain the complete loss of neurotransmitter release. Moreover, amperometry of neuroendocrine cell exocytosis along with overexpression of Munc18a and selected mutants showed that Munc18a has an effect on the initial release rate (15).

So far, there is little evidence from reconstitution experiments that Munc18a plays a major role in fusion itself, although stimulatory effects by SM proteins on SNARE-dependent lipid mixing were observed using ensemble fluorescence assays (16–18). A stimulatory effect of Munc18a was also observed in conjunction with neuronal SNAREs using a single vesicle-vesicle lipid-mixing assay (19). However, all of these experiments only used neuronal SNAREs in addition to Munc18a. Subsequent ensemble experiments that reconstituted neuronal SNAREs in liposomes along with neuron-specific proteins synaptotagmin-1 and complexin-1 showed only a relatively small increase of spontaneous (*i.e.* at zero Ca^2+^ concentration) lipid mixing and content mixing upon Munc18a inclusion when complexin-1 was present (see Fig. 3 (*D* and *F*) in Ref. 20).

Reconstitution experiments with a different subset of synaptic proteins showed that Munc18a acts in conjunction with NSF and αSNAP (soluble NSF adaptor protein), locking syntaxin-1A in a closed conformation and setting the stage for ternary *trans*-neuronal SNARE complex by the action of Munc13 and an approaching vesicle with synaptobrevin-2 (8). In this reconstitution experiment, Munc18a played an essential role to facilitate *trans*-SNARE complex formation in conjunction with NSF, αSNAP, and Munc13. Although an important step toward understanding the mechanism of Munc18, this study did not provide direct evidence that Munc18a stimulates fusion itself, because the amount of ensemble lipid mixing was roughly the same for a minimal system consisting only of neuronal SNAREs, the C2AB fragment of synaptotagmin-1, and 0.5 μm Ca^2+^ as well as for the more complete system consisting of the same proteins plus NSF, αSNAP, Munc18a, and Munc13 (see Fig. 4*D* in Ref. 8). However, it is possible that the ensemble lipid-mixing method used for this particular comparison may have masked an effect of Munc18a by opposing effects on the association of vesicles prior to fusion and fusion itself because ensemble fluorescence assays generally cannot distinguish between these two effects (21).

The function of SM proteins may be similar or different in other systems. As a case in point, Wickner and co-workers (22) showed that in the yeast vacuolar fusion system, efficient fusion with reconstituted proteins requires the HOPS complex, which contains the SM protein Vps33. HOPS, with its SM subunit, appears to be involved primarily in tethering and in a proofreading mechanism, rather than fusion itself (23–25), because its function can be replaced by synthetically induced tethering of vacuolar vesicles (26). However, the HOPS tethering complex causes a much more significant increase in the rate of fusion that cannot be explained by a simple increase in the number of *trans*-SNARE complexes (27), suggesting that it makes SNARE complexes more fusion-competent *prior to* fusion (*e.g.* by preventing transient antiparallel SNARE associations (28)).

Munc18a interacts with neuronal SNARE proteins throughout the synaptic vesicle cycle in complexes involving syntaxin-1A and the ternary SNARE complex (syntaxin-1A·SNAP-25·synaptobrevin-2) (29–31). Moreover, Munc18a co-localizes with syntaxin-1A and SNAP-25 in fixed cultured neurons and forms nanoclusters in the plasma membrane (32); knockdown of both syntaxin isoforms, syntaxin-1A and syntaxin-1B, disrupts the co-localization of Munc18a to SNAP-25.

Munc18a tightly binds to syntaxin-1A in a “closed” conformation in which its Habc domain associates in *cis* with its SNARE domain (29, 33), which in turn hinders the SNARE complex assembly (31, 34–36). Interestingly, in neuroendocrine cells, transfection of syntaxin alone results in its mislocalization, whereas co-transfection of both Munc18a and syntaxin results in trafficking to the plasma membrane (37, 38). Thus, one function of Munc18a may be to stabilize or “chaperone” syntaxin, which also agrees with the aforementioned knock-out studies of Munc18a in mice that showed a reduced level of syntaxin-1 in the plasma membrane (13).

Binding of Munc18a to the closed conformation of syntaxin-1A is not always required because rescue experiments in cultured syntaxin-1A-deficient neurons showed that the syntaxin-1A Habc domain is not required for evoked synaptic vesicle fusion, although it was required for spontaneous synaptic vesicle fusion (39). In contrast, the N-terminal residues of syntaxin-1A are required for binding of the ternary SNARE complex to Munc18a and may thereby serve as a recruiting factor for Munc18a (18). Because the Habc domain is needed for the closed conformation of syntaxin-1A and the tight interaction with Munc18a, this study suggests that the N-terminal and Habc domains mediate different functions in synaptic vesicle fusion.

In this paper, we start from the observation that a minimal system consisting of neuronal SNAREs and the soluble C2AB fragment of synaptotagmin-1 exhibited the same amount of lipid mixing as a more complete reconstitution that also included Munc18a, NSF, αSNAP, and Munc13 (8). At variance with that work, here we used full-length synaptotagmin-1 and included complexin-1, in a single vesicle-vesicle content-mixing assay. We recently used this assay to decipher the functions of complexin-1 (40) and found that it qualitatively reproduces effects observed in neuronal cultures for both spontaneous and Ca^2+^-triggered release for a variety of complexin-1 truncations and mutations, lending credence to this approach to investigate mechanistic questions.

In the context of studying the potential effect of Munc18a in vesicle fusion, our single vesicle content-mixing assay enables us to distinguish between fusion and vesicle association (often called “docking,” but we prefer not to use this term because it has a different meaning during the life cycle of a synaptic vesicle (1)). We investigated the effects of Munc18a on fusion and vesicle association between synaptic vesicle-mimicking vesicles (with reconstituted synaptobrevin-2 and synaptotagmin-1, referred to as SV vesicles) and plasma membrane-mimicking vesicles (with reconstituted syntaxin-1A and SNAP-25, referred to as PM vesicles) in the presence of complexin-1. We then used a single molecule assay to demonstrate that Munc18a is capable of sequestering syntaxin-1A from preformed t-SNARE complex. We also tested a double mutant that mimics phosphorylation of Munc18a(S306E/S313E) (41–44) as well as the role of the N-terminal residues of syntaxin-1A. To understand the molecular basis of the effect of Munc18a on vesicle association and fusion, we used purified proteins to demonstrate that Munc18a can interact specifically with the neuronal binary (syntaxin-1A·SNAP-25) SNARE complex (also referred to as t-SNARE complex) as well as the syntaxin-1A·SNAP-25·synaptobrevin-2 (ternary) SNARE complex.

## EXPERIMENTAL PROCEDURES

### Protein Purification

#### 

##### Synaptotagmin-1

Full-length rat synaptotagmin-1 (Syt1) was expressed in *Escherichia coli* from plasmid pJ414 (DNA 2.0, Menlo Park, CA) with a C-terminal decahistidine tag proceeded by a PreScission protease cleavage site to remove the tag and all cysteine residues changed to alanine except for the cysteine residue at position 277. Syt1 was expressed in *E. coli* BL21 (DE3) by growing the cells to *A*_600_ of 0.6–0.8 at 37 °C and then induced at 20 °C for 12–16 h with 0.5 mm isopropyl β-d-1-thiogalactopyranoside (IPTG). The cells from 6 liters of induced culture were harvested and suspended in 200 ml of synaptotagmin buffer A (50 mm sodium phosphate, pH 7.4, 600 mm NaCl, 2 mm DTT, and 10% glycerol (w/v)) supplemented with phenylmethylsulfonyl fluoride (PMSF) to 1 mm and two EDTA-free Complete Protease Inhibitor Mixture tablets (Roche Applied Science). Cells were lysed by three passes through the Emulsiflex C5 homogenizer (Avestin, Ottawa, Canada) at 15,000 p.s.i. Cell debris and inclusion bodies were removed by centrifugation at 8,000 rpm in a Beckman JA-20 rotor (Beckman Coulter, Brea, CA) for 10 min. The supernatant was then centrifuged at 8,000 rpm for 10 min in the same rotor. Membranes were collected by centrifugation at 40,000 rpm in a Beckman Ti-45 rotor (Beckman Coulter) for 1 h. Membranes were washed by homogenization in 100 ml of synaptogamin buffer A (50 mm sodium phosphate, pH 7.4, 600 mm NaCl, 2 mm DTT, 1 mm PMSF, 10% glycerol (w/v)) supplemented with two EDTA-free Complete Protease Inhibitor Mixture tablets (Roche Applied Science). Membranes were harvested again by centrifugation in a Ti-45 rotor (Beckman Coulter) at 40,000 rpm for 1 h. The pellet was suspended in 100 ml of synaptotagmin buffer B supplemented with 2 EDTA-free Complete Protease Inhibitor Mixture tablets and snap frozen in two 50-ml aliquots in liquid nitrogen. One aliquot of membranes was solubilized in the presence of 1.5% dodecylmaltoside (Anatrace, Maumee, OH) for 1 h at 4 °C. The extract was clarified by centrifugation in a Ti 45 rotor (Beckman Coulter) at 40,000 rpm for 35 min. The supernatant was bound to a 4-ml bed volume of Ni-NTA beads (Qiagen, Hilden, Germany) equilibrated in synaptotagmin buffer A containing 1.5% dodecylmaltoside (Anatrace) by stirring at 4 °C for 1 h. Beads were harvested by centrifugation and poured into a column, attached to an AKTA Prime FPLC system (GE Healthcare), and washed with 50 ml of synaptotagmin buffer A supplemented with 110 mm *n*-octyl-β-d-glucopyranoside (OG) (Anatrace) and 10 mm imidazole and then eluted with synaptotagmin buffer A supplemented with 110 mm OG and 500 mm imidazole. Protein-containing fractions were combined and injected on a Superdex 200 (16/60) column (GE Healthcare) equilibrated in 20 mm sodium phosphate, pH 7.4, 300 mm NaCl, 2 mm DTT, 110 mm OG, 10% glycerol (w/v) (synaptotagmin buffer B). Peak fractions were then combined, and 100 μg of prescission protease (GE Healthcare) was added to cleave the tag. Prescission protease-cleaved Syt1 was diluted with 20 mm sodium phosphate, pH 7.4, 2 mm DTT, 110 mm OG, 20 μm EGTA, 10% glycerol (w/v) (synaptotagmin buffer C) to bring the NaCl to 100 mm and then injected on a MonoS 5/50 column (GE Healthcare), washed with synaptotagmin buffer C containing 100 mm NaCl, and eluted with synaptotagmin buffer C containing 600 mm NaCl. Protein-containing fractions were dialyzed against synaptotagmin buffer C to lower the salt concentration and used for reconstitution into proteoliposomes.

##### Complexin-1

Full-length wild type complexin-1 (Cpx) was expressed in *E. coli* using BL21 (DE3) cells with a thrombin-cleavable N-terminal hexahistidine tag from plasmid pET28a (Novagen). Cells were grown in Luria Broth to an *A*_600_ between 0.6 and 0.8, and protein expression was induced by the addition of IPTG to 0.5 mm for 12–16 h at 20 °C. Cells were harvested by centrifugation and resuspended in 20 mm HEPES, pH 7.5, 500 mm NaCl, 2 mm DTT, 10 mm imidazole (complexin buffer A) supplemented with 1 mm PMSF and EDTA-free Complete Protease Inhibitor Mixture tablets (Roche Applied Science). Cells were lysed by passing them through the Emulsiflex C5 homogenizer (Avestin) at 15,000 p.s.i. three times. Lysate was clarified by centrifugation in a Ti-45 rotor (Beckman Coulter) for 35 min at 40,000 rpm. Supernatant was bound to a 4-ml bed volume of Ni-NTA beads (Qiagen) in batch, stirring for 1 h at 4 °C. The beads were harvested by centrifugation and poured into a column, attached to an AKTA Prime system (GE Healthcare), and washed with 60 ml of complexin buffer A containing 25 mm imidazole and then eluted with complexin buffer A containing 450 mm imidazole. Protein-containing fractions were combined, and 100 units of thrombin were added. This was then dialyzed against 500 ml of 20 mm HEPES, pH 7.5, 50 mm NaCl, 4 mm DTT (complexin buffer B) overnight at 4 °C. PMSF was added to 1 mm to quench the thrombin activity, and the mixture was injected onto a MonoQ column 5/50 (GE Healthcare) equilibrated with complexin buffer B. The column was washed with 20 column volumes of complexin buffer B, and the protein eluted with a linear NaCl gradient in complexin buffer B from 50 to 500 mm NaCl over 30 column volumes. The protein-containing fractions were combined and dialyzed against 20 mm HEPES, pH 7.5, 100 mm NaCl, 4 mm DTT overnight at 4 °C. The protein concentration was measured by absorbance at 280 nm, and aliquots were frozen in liquid nitrogen.

##### Wild Type Munc18a and Munc18a(S306E/S313E)

Wild type Munc18-1a (referred to here as Munc18a) and Munc18a (S306E/S313E) were expressed in *E. coli* with an N-terminal, TEV protease-cleavable hexahistidine tag from plasmid pPROExHTa. Proteins were expressed by the addition of IPTG to late log phase cultures (*A*_600_ ∼0.6–0.8) to 0.5 mm at 20 °C for 16–18 h. Cell pellets from 6 liters of culture were resuspended in 200 ml of Munc18a lysis buffer (20 mm sodium phosphate, pH 8, 300 mm NaCl, 10 mm imidazole), supplemented with 4 EDTA-free Complete Protease Inhibitor Mixture tablets (Roche Applied Science) and 1 mm PMSF and lysed by passing them three times through an Emulsiflex C5 homogenizer (Avestin) at 15,000 p.s.i. three times. The lysate was clarified in the Ti45 rotor (Beckman Coulter) at 40,000 rpm for 35 min. The supernatant was applied to a 5-ml Ni-NTA-agarose column (Qiagen), washed on an ATKA Prime FPLC system (GE Healthcare) with 100 ml of Munc18a lysis buffer supplemented with 50 mm imidazole, and then eluted with Munc18a lysis buffer supplemented with 300 mm imidazole. Protein-containing fractions were supplemented with 1 mm EDTA and 5 mm DTT, 100 μg of TEV protease was added to the combined protein-containing fractions to remove the hexahistidine tag, and the mixture was dialyzed overnight against 1 liter of 20 mm HEPES, pH 7.5, 50 mm NaCl, 1 mm EDTA, 5 mm DTT (monoQ buffer A). The TEV protease-cleaved Munc18a was then applied to a MonoQ 5/50 column (GE Healthcare) equilibrated in monoQ buffer A, and the column was eluted in a linear gradient from 50 to 500 mm NaCl. Protein-containing fractions were dialyzed against 20 mm HEPES, pH 7.5, 180 mm NaCl, 20 μm EGTA, 0.1% β-mercaptoehanol for 3 h and used immediately afterward in fusion and docking assays.

##### Full-length Syntaxin-1A and Synaptobrevin-2

Full-length rat syntaxin-1A (Stx1a) and synaptobrevin-2 were prepared as described previously (45). These proteins were expressed separately in *E. coli* with an N-terminal, TEV protease-cleavable, hexahistidine tag from plasmid pTEV5 (46). Proteins were expressed overnight at 25 °C in autoinducing medium (47) in *E. coli* strain C43 (48). Cell pellets from 8 liters of culture were suspended in 400 ml of 50 mm sodium phosphate, pH 8, 1 m NaCl, 5 mm EDTA, and 1 mm PMSF supplemented with Complete Protease Inhibitor Mixture tablets (Roche Applied Science) and broken by three passes through a M-110-EH microfluidizer (Mircrofluidics Corp., Newton, MA) at 15,000 p.s.i. Inclusion bodies were removed by two consecutive 10-min spins at 10,000 rpm in a JA-14 (Beckman Coulter) rotor, and the membrane fraction was collected by centrifugation at 40,000 rpm for 2 h in a Ti-45 (Beckman Coulter) rotor. Membranes containing syntaxin-1A were further washed with a buffer containing 10 mm Tris-H_2_SO_4_, pH 7.5, 10 mm EDTA, 10% glycerol (w/v), centrifuged at 40,000 rpm for 1 h in a Ti-45 rotor. Membrane pellets were resuspended in 20 mm HEPES, pH 7.5, 500 mm NaCl, 1 mm tris(2-carboxyethyl)phosphine (TCEP), and 10% glycerol (w/v) and centrifuged for an additional 1 h in the same rotor. Membranes were suspended to a concentration of 5 mg/ml in 20 mm HEPES, pH 7.5, 500 mm NaCl, 1 mm TCEP, 10 mm imidazole, and 10% glycerol (w/v), 1 mm PMSF, and EDTA-free Complete Protease Inhibitor Mixture. Dodecylmaltoside (Anatrace) was added to 2% (w/v), and after incubation at 4 °C for 1 h, the sample was centrifuged for 35 min at 40,000 rpm in a Ti-45 (Beckman Coulter) rotor, and the supernatant was loaded onto a 1-ml column of Ni-NTA-agarose (Qiagen). The column was washed with 20 mm HEPES, pH 7.5, 300 mm NaCl, 1 mm TCEP, 20 mm imidazole, 110 mm OG, and 10% glycerol (w/v), and the proteins were eluted in the same buffer containing 450 mm imidazole and 1 m NaCl. 1 mm EDTA was immediately added to the pooled fraction and loaded onto a Superdex 200 HR 10/300 GL column (GE Healthcare) that was equilibrated with 20 mm HEPES, pH 7.5, 300 mm NaCl, 1 mm TCEP, 110 mm OG, and 10% glycerol (w/v). Protein fractions were pooled and digested with 100 μg TEV protease for 30 min at ambient temperature, after which the TEV protease had precipitated. TEV was removed by centrifugation at 5,000 rpm for 10 min in an Eppendorf model 5804 R tabletop centrifuge (Eppendorf North America, Hauppauge, NY). The sample in the supernatant was again digested with 100 μg of TEV protease for 30 min at ambient temperature, after which the reaction was complete and the TEV protease had precipitated. TEV was removed by centrifugation at 5,000 rpm for 10 min in the same rotor.

**FIGURE 1.**
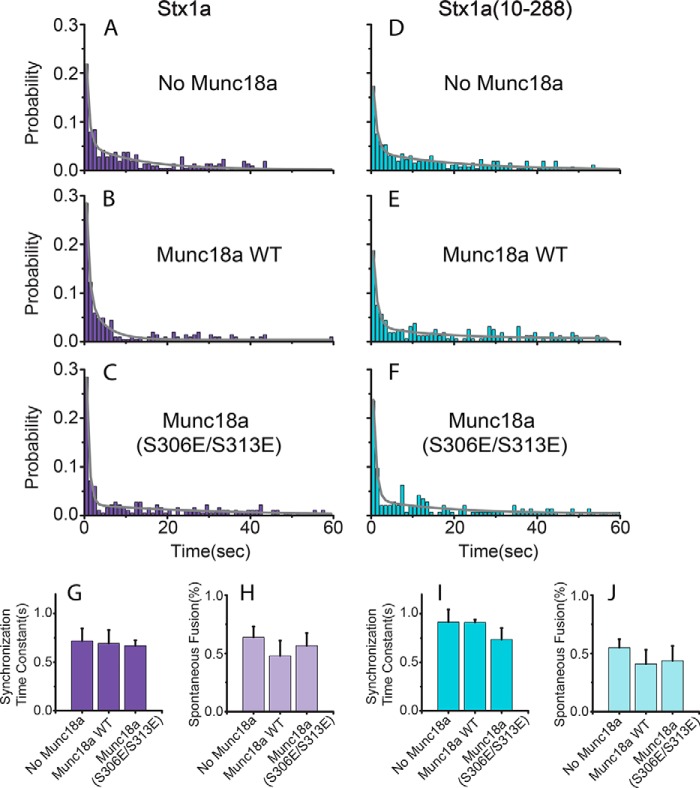
**Effect of Munc18a on Ca^2+^-triggered fusion and spontaneous fusion between SV and PM vesicles after 30-min incubation of PM vesicles with Munc18a.** *A–F*, histograms of Ca^2+^-triggered fusion events, without Munc18a or with Munc18a or Munc18a(S306E/S313E), using PM vesicles with reconstituted full-length Stx1a or Stx1a(10–288), as specified in the *panels*. All experiments were performed in the presence of 2 μm complexin. The histograms were calculated with a bin size of 1 s, normalized by the total of fusion events observed upon Ca^2+^ injection, and fitted to two-exponential decay functions (*solid lines*). The synchronization time constants (faster time constant of the fitted two-exponential fit) are shown in *G*. Spontaneous fusion events were observed for 1 min before Ca^2+^-injection and shown in *H. Error bars* in *G*, 1σ errors computed from the covariance matrix during least square fitting using a Levenberg-Marquardt technique; *error bars* in *H*, S.E. values over at least eight experiments.

The N-terminally truncated construct of syntaxin-1A (Stx1a (10–288)) was expressed with an N-terminal thrombin-cleavable glutathione *S*-tranferase tag from plasmid pGEX-KG (49). After induction with 0.1 mm IPTG at *A*_600_ of 0.8, the proteins were expressed overnight at 16 °C at 100 rpm in Luria broth in BL21 (DE3) *E. coli* cells (Novagen, EMD Chemicals, Gibbstown, NJ). Cell pellets from 1 liter of culture were suspended in 40 ml of sodium phosphate, pH 8, 1 mm dithiothreitol, 0.2% Triton X-100, 1% dodecylmaltoside, and 0.2 mm PMSF supplemented with Complete Protease Inhibitor Mixture tablets (Roche Applied Science), and the cells were disrupted by sonication at 5/15-s on/off pulse for 5 min at 50% power using a Sonicator Ultrasonic Processor XL-2020 (Misonix, Farmingdale, NY). Inclusion bodies were removed by a 10-min 15,000-rpm spin in a JA-20 (Beckman Coulter) rotor and further cleared by centrifugation at 40,000 rpm for 35 min in a Ti-45 (Beckman Coulter) rotor. The supernatant was incubated for 2 h with a 1.5-ml slurry of glutathione-Sepharose 4 Fast Flow beads (GE Healthcare) that was pre-equilibrated in the same buffer. The beads were then collected in a column and washed with 80 column volumes of buffer containing 50 mm sodium phosphate, pH 8, 1 mm dithiothreitol, and 0.2% Triton X-100 and then with 30 column volumes of buffer containing 40 mm Tris-HCl, pH 8, 150 mm NaCl, 1 mm dithiothreitol, and 0.8% OG. The beads were then incubated for 90 min at ambient temperature with 100 units of bovine α-thrombin (Hematologic Technologies, Essex Junction, VT) in buffer containing 40 mm Tris-HCl, pH 8, 150 mm NaCl, 1 mm dithiothreitol, and 3% OG. The supernatant containing cleaved ΔN syntaxin(10–288) was flowed off the column, after which the thrombin was quenched with 1 mm PMSF. Although the GST-syntaxin-1A construct was designed to include the N terminus of syntaxin-1A, the application of thrombin proteolysis also removed nine additional residues at the N terminus as determined by Edman sequencing. Removal of these nine N-terminal residues has no significant effect on fusion efficiency and kinetics for spontaneous and Ca^2+^-triggered fusion with neuronal SNAREs, synaptotagmin-1, and complexin-1 (Fig. 1, *G* and *H*).

##### SNAP-25 and SNAP-25(K76C) Constructs for Vesicle Reconstitution and Single Molecule Experiments

Cysteine-free SNAP-25 (C84S, C85S, C90S, and C92S) or single cysteine-containing SNAP-25(K76C) (C84S, C85S, C90S, C92S, and K76C) was expressed from plasmid pTEV5 (46) with an N-terminal TEV protease-cleavable hexahistidine tag. The proteins were expressed overnight in autoinducing medium (47) in *E. coli* strain BL21(DE3) at 30 °C. Cells from 4 liters of culture were resuspended in 200 ml of 50 mm sodium phosphate, pH 8.0, 300 mm NaCl, 20 mm imidazole, and 10% glycerol (w/v) supplemented with 1 mm PMSF and four EDTA-free protease inhibitor mixture tablets. Cells were lysed by three passes through an Emulsiflex C5 homogenizer (Avestin) at 15,000 p.s.i. The lysate was clarified by centrifugation in the Ti-45 rotor for 1.5 h at 40,000 rpm. The supernatant was bound to a 5-ml Ni-NTA column by flowing the lysate onto the column at 1 ml/min using an AKTA Prime FPLC system (GE Healthcare). The column was washed with 150 ml of SNAP-25 buffer containing 50 mm sodium phosphate, pH 8.0, 300 mm NaCl, 10% glycerol (w/v), supplemented with 50 mm imidazole, and eluted with buffer containing 50 mm sodium phosphate, pH 8.0, 300 mm NaCl, 10% glycerol (w/v), and 350 mm imidazole. Protein-containing fractions were combined, DTT was added to 5 mm, EDTA was added to 1 mm, and 150 μg of TEV protease was added to remove the hexahistidine tag. This mixture was dialyzed against buffer containing 20 mm HEPES, pH 7.5, 100 mm NaCl, 4 mm DTT, and 10% glycerol (w/v) overnight at 4 °C. The TEV-cleaved SNAP-25 was concentrated in a 15-ml Amicon ultracentrifugal concentrator with a 10,000 molecular weight cut-off dialysis cassette (Millipore, Billerica, MA) to 5 ml and injected onto the Superdex 200 (16/60) column (GE Healthcare) equilibrated with buffer containing 20 mm HEPES, pH 7.5, 100 mm NaCl, 4 mm DTT, 10% glycerol (w/v). Protein-containing fractions were combined and then concentrated using a 3,000 molecular weight cut-off dialysis cassette (Millipore). The concentration of SNAP-25 was measured by absorbance at 280 nm, and aliquots were frozen in liquid nitrogen.

Purified SNAP-25(K76C) was incubated with a 10-fold molar excess of TCEP for 10 min followed by incubation with a 10-fold molar excess of Alexa647 c2 maleimide at room temperature for 2 h. The reaction mix was then transferred to 4 °C overnight. A Sephadex G-50 column (Sigma-Aldrich) was used for crude separation of free Alexa647 from proteins. An additional dialysis step was done using a 10,000 molecular weight cut-off dialysis cassette (Millipore) at 4 °C against buffer containing 20 mm HEPES, pH 7.5, 90 mm NaCl to remove remaining free dye. The concentrations of SNAP-25(K76C) and Alexa647 were measured by absorbance at 280 and 651 nm, and aliquots were frozen in liquid nitrogen.

##### Soluble Syntaxin-1A and Synaptobrevin-2 Constructs and Soluble t-SNARE Complex

N-terminally GST-tagged SNAP-25 (amino acids 1–206) and the cytoplasmic domain of synaptobrevin-2(1–96) (amino acids 1–96) were expressed from a pGEX vector (GE Healthcare), and Munc18a with an N-terminal His_6_ tag was cloned into a pProExHTa vector (Life Technologies, Inc.). Cytoplasmic syntaxin-1a constructs with residues 1–267 (Stx1a(1–267)) and residues 25–267 (Stx1a(25–267)) were expressed from the pPAL7 vector with an N-terminal Profinity eXact tag (Bio-Rad). The t-SNARE complex was produced by co-expressing N-terminally His_6_-tagged SNAP-25 with Stx1a(1–267) lacking an affinity tag from a pET-Duet vector (Novagen). All recombinant proteins were expressed in *E. coli* BL21 (DE3) RIL+ in LB medium at 37 °C to an *A*_600_ of 0.6–0.8, induced with 1 mm IPTG, and grown for an additional 4–5 h at 21–37 °C. Pellets were resuspended in lysis buffers optimized for affinity purification with protease inhibitor mixture set V (Calbiochem) and DNase I (Sigma). Standard lysis buffer (20 mm Tris, pH 8.0, 300 mm NaCl, 1 mm EDTA, 1 mm DTT) was used for GST fusion proteins, TALON buffer (20 mm Na_2_HPO_4_/NaH_2_PO_4_, pH 8.0, 300 mm NaCl, 5 mm imidazole) for His-tagged proteins, and Profinity buffer (100 mm Na_2_HPO_4_/NaH_2_PO_4_, pH 8.0) for Stx1a(1–267). Cells were lysed using an Emulsiflex (Avestin) at a maximum pressure of 15,000 p.s.i., and lysates were centrifuged at 39,000 relative centrifugal force for 30 min at 4 °C to remove insoluble material. Clarified lysates were incubated with equilibrated glutathione-agarose beads, TALON metal affinity resin (Clontech), or Profinity eXact beads (Bio-Rad) and washed with the respective lysis buffers. Proteins were eluted from glutathione-agarose beads with 20 mm reduced glutathione and from TALON resin with 200 mm imidazole or cleaved from Profinity resin following a 2-h incubation at room temperature with 100 mm NaF, yielding Stx1a(1–267) with a native N terminus. GST fusion proteins were combined with thrombin (90 units) and dialyzed against cleavage buffer (20 mm Tris, pH 8.0, 150 mm NaCl, 10 mm DTT, 2.5 mm CaCl_2_) overnight at 4 °C. All proteins (except synaptobrevin-2(1–96)) were dialyzed against low salt buffer (20 mm Tris, pH 8.0, 75 mm NaCl, 1 mm EDTA, and 1 mm DTT) for 2 h at 4 °C, purified by fast protein liquid chromatography on HiTrapQ or MonoQ columns (GE Healthcare), and eluted with a linear NaCl gradient. Cleaved synaptobrevin-2(1–96) was dialyzed against 20 mm MES, pH 6.5, 75 mm NaCl, 1 mm EDTA, and 1 mm DTT for 2 h at 4 °C and loaded on a MonoS column (GE Healthcare) to separate thrombin and GST from synaptobrevin-2(1–96). For isothermal titration calorimetry (ITC), the t-SNARE complex produced by co-expression (see above) was further purified on a preparative S200 column (GE Healthcare) in 20 mm Tris, pH 8.0, 150 mm NaCl, 1 mm EDTA, and 1 mm DTT. Peak fractions containing equal-intensity bands were pooled to maximize complex containing 1:1 Stx1a·SNAP-25. Ternary SNARE complex for ITC was formed by combining a 1.5:1 molar excess of synaptobrevin-2(1–96)·t-SNAREs, dialyzing against low salt buffer overnight at 4 °C, and purifying on a MonoQ column. Peak fractions were pooled and concentrated. Munc18a was added in a slight molar excess to t-SNAREs and incubated overnight at 4 °C prior to the limited proteolysis experiment. For anisotropy, Munc18a·t-SNARE complex was purified on an analytical S200 gel filtration column.

##### Cytoplasmic Syntaxin-1A Constructs for Bio-layer Interferometry Experiments

Cytoplasmic syntaxin-1A constructs with residues 1–265 (Stx1a(1–265)) and residues 25–265 (Stx1a(25–265)) were fused with a C-terminal Avitag sequence (Avidity, Aurora, CO) replacing the transmembrane domain and cloned into the pTEV5 vector (46) that encodes an N-terminal TEV protease-cleavable hexahistidine tag. Biotinylation was conducted *in vivo* by expression in AVB101 cells (Avidity) and induced with 0.5 mm IPTG overnight at 25 °C in the presence of 50 μm biotin.

### Protein Reconstitution in Liposomes

We used the same membrane compositions and protein densities as in our previous studies (45, 50). Likewise, the reconstitution protocol was similar (50) with several changes; the concentration of 2-mercaptoethanol in the dialysis and vesicle-free buffers was changed from 1 to 0.1%, an extra dialysis step was added to ensure removal of Ca^2+^ and detergent, all dialysis buffers contained 0.8 g/liter Chelex 100 resin (Bio-Rad) and 2.5 g/liter Bio-beads SM2 (Bio-Rad), and the Sepharose CL-4B column (GE Healthcare) was packed under constant pressure.

Briefly, SV vesicles were reconstituted with both synaptotagmin-1 and synaptobrevin-2 (except when noted otherwise), whereas PM vesicles were reconstituted with syntaxin-1A and SNAP-25A, using the lipid compositions described previously. The protein/lipid ratios used were 1:200 for synaptobrevin and syntaxin and 1:800 for synaptotagmin. A 3–5-fold excess of SNAP-25 (with respect to syntaxin-1A) and 3.5 mol % PIP_2_ (unless noted otherwise) were added to the protein/lipid mixture for PM vesicles only; the excess ratio of SNAP-25 to syntaxin-1A essentially eliminates changes of formation of non-productive 2:1 syntaxin-1A·SNAP-25 complexes (51). Dried lipid films were dissolved in 110 mm OG buffer containing purified proteins. Detergent-free buffer (20 mm HEPES, pH 7.4, 90 mm NaCl, 0.1% 2-mercaptoethanol) was then added to the protein-lipid mixture until the detergent concentration reached the critical micelle concentration of 24.4 mm. The vesicles were then subjected to size exclusion chromatography using a Sepharose CL-4B column, packed under near constant pressure by gravity with a peristaltic pump (GE Healthcare) in a 5-ml column with a 2-ml bed volume that was equilibrated with buffer V (20 mm HEPES, pH 7.4, 90 mm NaCl, 20 μm EGTA, 0.1% 2-mercaptoethanol) followed by dialysis into 2 liters of detergent-free buffer V supplemented with 5 g of Bio-beads SM2 and 0.8 g/liter Chelex 100 resin. After 4 h, the buffer was changed with 2 liters of fresh buffer V containing Bio-beads and Chelex, and dialysis continued for 12 h. During the preparation of SV vesicles, 50 mm sulforhodamine B (Invitrogen) was present in all solutions prior to the size exclusion chromatography step. As described previously (51), the presence and purity of reconstituted proteins were confirmed by SDS-PAGE of the vesicle preparations, and the directionality of the membrane proteins (facing outward) was assessed by chymotrypsin digestion followed by SDS-PAGE. The size distributions of the SV and PM vesicles were analyzed by cryo-EM, as described previously (45).

### Single Vesicle-Vesicle Content-mixing Assay

After a surface passivation with polyethylene glycol (PEG) molecules to eliminate nonspecific binding of vesicles, a #1.5 glass coverslip (VWR International) was assembled into a flow chamber and coated with neutravidin (0.1 mg/ml) (50). PM vesicles at a 100× dilution were immobilized on the PEG-coated surface for a 30-min incubation period in buffer V in the presence or absence of 1 μm Munc18a (or mutant Munc18a(S306E/S313E)) at room temperature (∼25 °C). This incubation period was followed by three rounds of 150-μl buffer washes with buffer V along with Munc18a if it was present in the previous stage. SV vesicles were loaded into the flow chamber in buffer V along with 2 μm complexin-1 and Munc18a if it was present in the previous stage. For this loading step, the SV vesicle concentrations were adjusted to account for reduction of PM vesicle association when Munc18a was present during the incubation period; the PM vesicle stock solution was diluted 1,000 times in the absence of Munc18/Munc18a(S306E/S313E) or diluted 100 times in the presence of Munc18a or diluted 300 times in the presence of Munc18a(S306E/S313E). The system was incubated for 30 min, followed by three rounds of washes with buffer V supplemented with 2 μm complexin-1, and 1 μm Munc18a if it was present in the previous stage. Buffer V, supplemented with 500 μm Ca^2+^, 2 μm complexin, and 1 μm Munc18a if it was present in the previous stage, was injected into the flow chamber at a speed of 2 ml/min by a motorized syringe pump. The TTL signal feeding to the syringe pump was delayed by a fixed time with respect to the start of the movie recording by a 9520 digital delay pulse generator (Quantum Composers, Bozeman, MT). Prior to Ca^2+^ injection, we monitored spontaneous fusion events for a period of 1 min in the same channel that was also used for Ca^2+^ injection. Real-time traces with at least one florescent jump after the triggering were analyzed. The details of automated data analysis were reported in previous work (52). Histograms of Ca^2+^-triggered fusion events were calculated with a 1-s time bin and fitted to a two-exponential decay function. The faster time constant of the fitted two-exponential decay function is defined as the synchronization time constant of Ca^2+^-triggered fusion.

### Single Vesicle-Vesicle Association Experiments

This assay is similar to the single vesicle-vesicle content-mixing assay described above with some modifications in order to enable higher throughput. We followed a protocol for studying the association of single free SV vesicles with immobilized PM vesicles described previously (53, 54). A saturated layer of unlabeled PM vesicles was immobilized on an imaging surface via biotin/neutravidin interactions. Specifically, the PM vesicle solution was diluted 10 times with buffer V. 100 μl of the diluted PM vesicle solution was injected into the sample chamber and incubated for 30 min, followed by buffer exchange (1 × 200 μl of buffer V) for 6 s. Next, 100 μl of solution containing wild type or Munc18a mutant (1 μm) was injected into the sample chamber and incubated for 30 min, followed by buffer exchange (1 × 200 μl of buffer V) for 6 s. Subsequently, 100 μl of 500–1,000× diluted SV vesicle solution was injected into the flow chamber in the presence of 2 μm complexin-1. After an incubation period of 30 min, unbound SV vesicles were removed by buffer exchange (2 × 200 μl of buffer V for ∼20 s). 20 μm EGTA was included to ensure the absence of free Ca^2+^ ions. For the control with soluble synaptobrevin-2(1–96), an additional 30-min incubation period with 10 μm synaptobrevin-2(1–96) was included between the Munc18a incubation and SV vesicle incubation stages.

For the preassociated vesicle experiment, the Munc18a and the SV vesicle incubations were reversed with the same conditions: SV vesicles preassociated with PM vesicles for 30 min and then incubated in the flow chamber with or without Munc18a for 30 min. Associated SV vesicles were counted after one round of buffer wash (6 s) to remove any SV vesicles that may have become unbound by the action of Munc18a.

Sample slides with five channels were monitored in a wide field total internal reflection fluorescence microscope (Nikon) using an electron multiplying charge-coupled device (CCD) camera (iXon+ DV 897E, Andor Technology). A program (smCamera) written in C++ was used for data acquisition and analysis (available from Taekjip Ha, University of Illinois). 10 images were taken at random locations within each channel on the quartz slide. Details regarding software, slide assembly, and imaging protocols are as described previously (55).

As described in our previous work (53, 54), the PM vesicle-covered surfaces were saturated and produced a homogeneous distribution for each surface preparation with red laser excitation (633 nm) of the DiD-labeled immobilized vesicles. Our preparation of a reproducible, homogeneous, and saturated surface of immobilized PM vesicles ensures that the number of associated SV vesicles is directly related to the association probability. To minimize variation among different slides, for each set of comparisons between different conditions and/or mutants, the same protein preparations and surface preparations (quartz slide with immobilized vesicles) and incubation times were used, and the experiments were carried out in separate channels on the same slide. We found that the relative differences and ratios were statistically similar for different protein and surface preparations.

### Single Molecule SNAP-25 Experiments

In order to probe release of SNAP-25 from t-SNARE complex by Munc18a, PM vesicles were reconstituted with syntaxin-1A and a 100:1 mixture of unlabeled and labeled SNAP-25. For labeled SNAP-25, the protein was mutated at position 76 (K76C) and labeled with Alexa647. The reconstitution was performed using the same protocol as used for all other single vesicle experiments in this work. Likewise, the PM vesicles with the mixture of labeled and unlabeled SNAP-25 molecules were immobilized on an imaging surface via biotin/neutravidin interactions with the same protocol as used for all other single vesicle experiments in this work. Fluorescence intensity from single vesicles was monitored before and after the Munc18a addition; specifically, 1 μm Munc18a was injected into the chamber and incubated for 120 min, followed by buffer exchange (3 × 200 μl of vesicle buffer for ∼20 s). A slide with multiple channels was monitored in a wide field total internal reflection fluorescence microscope, and data were acquired with a CCD camera and analyzed with the smCamera program, similar to the single vesicle-vesicle association experiments described above. 10 images were taken at random locations within each channel on the quartz slide. The number of vesicles decorated with Alexa647-labeled SNAP-25 was determined by counting the number of Alexa647 fluorescent spots from emission of Alexa Fluor upon excitation at 633 nm before and after the Munc18a addition.

### Pull-down Assay

Purified Stx1a(1–267) and Stx1a(25–267) were incubated with GST or GST-SNAP-25 in phosphate-buffered saline (PBS) solution (20 mm Na_2_HPO_4_/NaH_2_PO_4_, pH 7.4, 150 mm NaCl, 1 mm DTT) overnight at 4 °C and then incubated with Munc18a for 1.5 h and subsequently combined with equilibrated glutathione-agarose beads (final protein concentration ∼7 μm) and incubated for an additional 1.5 h at 4 °C. Samples were spun at 850 relative centrifugal force for 5 min at 4 °C, followed by separation of supernatant and bead fractions. The latter were washed twice with 0.5 ml of PBS (spun at 850 relative centrifugal force for 5 min at room temperature), combined with SDS, boiled, and analyzed by SDS-PAGE.

### Isothermal Titration Calorimetry

ITC experiments were conducted using a VP-ITC instrument (Microcal) at 25 °C. Samples were dialyzed against 1 liter of PBS overnight at 4 °C, filtered, and degassed prior to each run. Munc18a (cell) concentrations ranged from 9 to 11 μm, t-SNARE and SNARE complex (syringe) concentrations ranged from 100 to 180 μm, and titration injection volumes varied from 7 to 10 μl, 35–45 injections total, with 8–24 s/injection and 4 min between injections. Microcal Origin version 7.0 software was used to integrate and analyze the measured heat released on binding. Fitting the data to a single site binding model gave the equilibrium association constant *K_a_*, the enthalpy of binding Δ*H*, and the stoichiometry *n*.

### Fluorescent Labeling of the Soluble Synaptobrevin-2 Fragment

Purified single cysteine soluble fragment of synaptobrevin-2(1–96) (S79C) was dialyzed against labeling buffer (20 mm HEPES, pH 7.0, 150 mm NaCl) overnight at 4 °C and subsequently incubated with excess Alexa488-maleimide (Life Technologies) for a molar ratio of 1:2 (synaptobrevin-2(1–96)/Alexa488) in 10 mm TCEP for 24 h at 4 °C. Labeled protein (synaptobrevin-2(1–96)-A488) was subsequently dialyzed against 20 mm MES, pH 6.0, 75 mm NaCl, 1 mm EDTA, and 1 mm DTT for 2 h at 4 °C and loaded on a MonoS column (GE Healthcare). Fractions containing labeled protein were pooled and concentrated.

### Fluorescence Anisotropy Experiments

All experiments were performed at 25 °C in PBS (200-μl reaction volume) in black, flat-bottomed 96-well plates (Corning, Inc.) using a Synergy4 Hybrid Multi-Mode Microplate reader (BioTek). SNAP-25 and cytoplasmic Stx1a(1–267) (0.25–5 μm) were combined in molar ratios of 1:1 or 1:1.5 (Stx1a(1–267)/SNAP-25). The amount of Munc18a (0.25–5 μm) added to cytoplasmic t-SNAREs was fixed at 1:1 or varied from 0.3 to 4:1 (Munc18/t-SNAREs). Pre-formed Munc18a·t-SNARE complexes were purified on an S200 gel filtration column. All proteins were in excess of synaptobrevin-2(1–96)-A488 (50 nm). BSA (0.1–0.6 mg/ml) was used to minimize nonspecific binding of synaptobrevin-2(1–96)-A488 to the plastic well walls. Proteins were incubated separately and/or together in wells for 0.5–2 h at room temperature, followed by a 10-min “warm-up” run in the plate reader at 25 °C. The final component (synaptobrevin-2(1–96)-A488 or t-SNARE(s)) was added to start the reaction. Fluorescence anisotropy measurements were recorded every 20–30 s for 40–50 min. Anisotropy (*r*) was calculated using the equation, *r* = (*I*_VV_ − *GI*_VH_)/(*I*_VV_ + 2*GI*_VH_), where *G* = *I*_HV_/*I*_HH_, *I* is fluorescence intensity, the first subscript letter represents the direction of excitation light, and the second subscript letter represents the direction of emission light for horizontally (H) and vertically (V) polarized light. Baselines were subtracted from all spectra, and data were analyzed using Prism version 5.0 (GraphPad Software). This anisotropy assembly assay was validated in Ref. 31, where it was shown that tagged synaptobrevin-2 assembles into an SDS-resistant complex, which is the gold standard for an assembled, stable neuronal SNARE complex.

### Bio-layer Interferometry

Binding affinities between Stx1a(1–265) or Stx1a(25–265) and Munc18a or Munc18a(S306E/S313E) were measured using bio-layer interferometry in an Octet QK system equipped with Streptavidin-coated biosensor tips (ForteBio, Menlo Park, CA). Analyses were performed in 20 mm HEPES, pH 7.5, 90 mm NaCl, 20 μm EGTA, 0.1% β-mercaptoethanol, 0.05 mg/ml BSA at 30 °C using 96-well microplates (Greiner) shaking with a speed of 1,000 rpm during all experimental steps. Streptavidin biosensor tips were loaded over 10–30 s or up to a binding height of ∼1 nm in Stx1a(1–265) or Stx1a(25–265) containing a C-terminal Avitag sequence. To monitor complex formation between Munc18a and the syntaxin-1A constructs, the syntaxin-1A-loaded tips were dipped into a Munc18a dilution series (0, 5, 10, 20, 40, 80, 160, and 320 nm for WT Munc18a and Munc18a(S306E/S313E)) and allowed to associate for 200–1,000 s. Dissociation (200–1,000 s) was performed in buffer (20 mm HEPES, pH 7.5, 90 mm NaCl, 20 μm EGTA, 0.1% β-mercaptoethanol, 0.05 mg/ml BSA).

## RESULTS

### 

#### 

##### Munc18a Has No Effect on Fusion Kinetics

We used our single vesicle-vesicle content-mixing assay to study both spontaneous and Ca^2+^-triggered fusion (50, 51) with modifications to protein preparation, reconstitution, and single vesicle imaging as described under “Experimental Procedures.” Syntaxin-1A and SNAP-25 were reconstituted in PM vesicles decorated with biotin-PE lipids for surface immobilization. Synaptobrevin-2 and synaptotagmin-1 were reconstituted in “SV” vesicles that contained encapsulated sulforhodamine B as a content indicator. PM vesicles were then immobilized on the surface of a flow chamber and incubated with or without Munc18a for 30 min. SV vesicle solution was then injected into the flow chamber in the presence of complexin-1; Munc18a was included if it had been present in the previous incubation stage. Ca^2+^ solution was then injected into the sample chamber using the same buffer, which included complexin-1 and/or Munc18a if it had been present in the previous stage. Prior to Ca^2+^ injection, spontaneous fusion events were monitored for a 1-min period. Upon Ca^2+^ injection, fusion events were monitored, and time delays of fusion events with respect to the time point of Ca^2+^ injection were plotted as binned histograms (Fig. 1, *A–F*).

**FIGURE 2.**
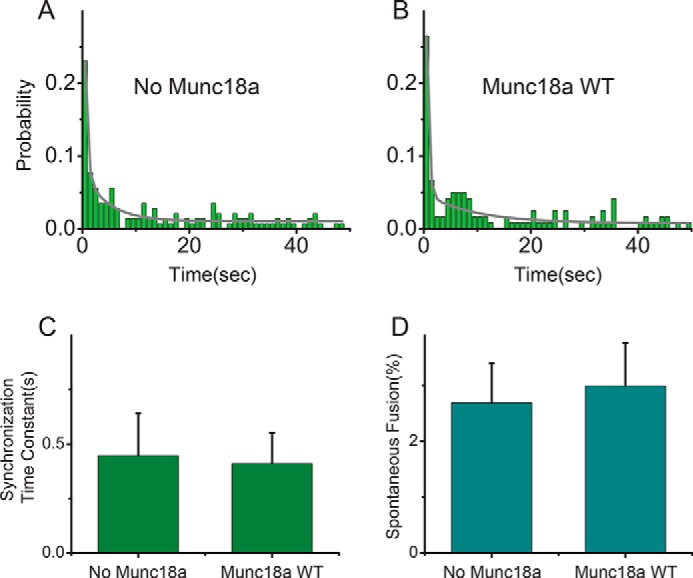
**Effect of Munc18a on Ca^2+^-triggered fusion and spontaneous fusion with concurrent addition of Munc18a and SV vesicles to immobilized PM vesicles.** Shown are histograms of Ca^2+^-triggered fusion events without Munc18a (*A*) and with 1 μm Munc18a (*B*) using PM vesicles containing full-length Stx1a. Munc18a and SV vesicles were introduced concurrently. All experiments were performed in the presence of 2 μm complexin. The histograms were calculated with a bin size of 1 s, normalized by the total of fusion events observed upon Ca^2+^ injection, and fitted to two-exponential decay functions (*solid lines*). The synchronization time constants (faster time constant of the fitted two-exponential fit) are shown in *C*. Spontaneous fusion events were observed for 1 min before Ca^2+^ injection and shown in *D. Error bars* in *C*, are 1σ errors computed from the covariance matrix during least square fitting using a Levenberg-Marquardt technique; *error bars* in *D*, S.E. value over 4–6 experiments.

Munc18a had no effect on the normalized Ca^2+^-triggered fusion histograms (Fig. 1, *A–F*), the synchronization time constant (Fig. 1*G*), or the probability of spontaneous fusion (Fig. 1*H*), regardless of whether full-length Stx1a or the N-terminally truncated Stx1a(10–288) fragment were used. Moreover, there was no effect if Munc18a was incubated concurrently when adding free SV vesicles to the immobilized PM vesicles (Fig. 2). We note that the histograms were normalized with respect to the total number of fusion events, so they measure the effect of Munc18a on the intrinsic spontaneous and Ca^2+^-triggered fusion kinetics of associated vesicle-vesicle pairs. The ability to measure intrinsic fusion kinetics is an advantage compared with ensemble fusion experiments, where effects on docking and fusion cannot be separated (21).

##### Munc18a Binds to the Binary t-SNARE Complex

**FIGURE 3.**
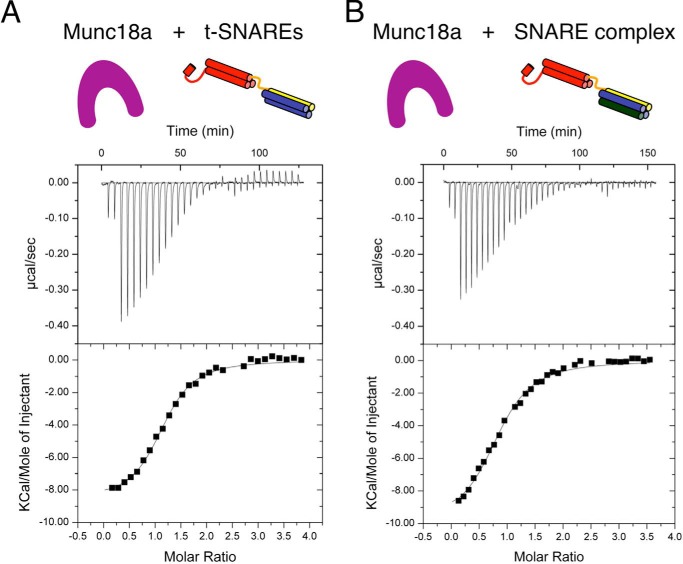
**Munc18a binds soluble t-SNAREs and SNARE complex with similar thermodynamics.** t-SNAREs (*A*) or SNARE complex (100–180 μm) (*B*) was titrated into Munc18a (9–11 μm). The *top panel* shows the heat signals corresponding to each injection. The *bottom panel* shows the integrated areas *versus* molar ratio of interacting species. Data were fit to a single binding site model using a nonlinear least squares fit (*solid line*). Thermodynamic parameters are presented in Table 1.

Our vesicle fusion assay uses PM vesicles that bear assembled Stx1a·SNAP-25 binary (t-SNARE) complexes, so we asked whether Munc18a could bind to the t-SNARE complex. We used ITC to measure the binding thermodynamics of Munc18a to the soluble t-SNARE complex. For direct comparison, we carried out ITC measurements of Munc18a-binding to the soluble ternary neuronal SNARE complex, as described previously (31). Control experiments in which t-SNAREs or SNARE complex were injected into buffer confirm that both complexes remain intact over the course of an ITC run (*i.e.* no dissociation occurred upon dilution). Remarkably, the binding energetics of Munc18a to the t-SNARE complex and to the ternary SNARE complex are very similar (Fig. 3 and Table 1). However, the affinity for both complexes is much weaker than for syntaxin-1A alone (*K_d_* of 1.4 nm (31, 56)).

**TABLE 1:** **Thermodynamic parameters of Munc18a interaction with t-SNARE and ternary SNARE complexes** Our results differ slightly from previously published ITC data for Munc18a-binding SNARE complex (see supplemental Table S1 in Ref. 31), which probably reflects differences in the protein constructs and preparations.

Munc18a interaction partner	*K_d_*	Δ*H*^0^	*n*
	μ*m*	*kcal/mol*	
Stx1a(1–267) in t-SNARE complex	1.2 ± 0.5	−8.3 ± 0.9	1.4
Stx1a(1–267) in ternary SNARE complex	1.9 ± 0.7	−10.5 ± 0.6	0.8

**FIGURE 4.**
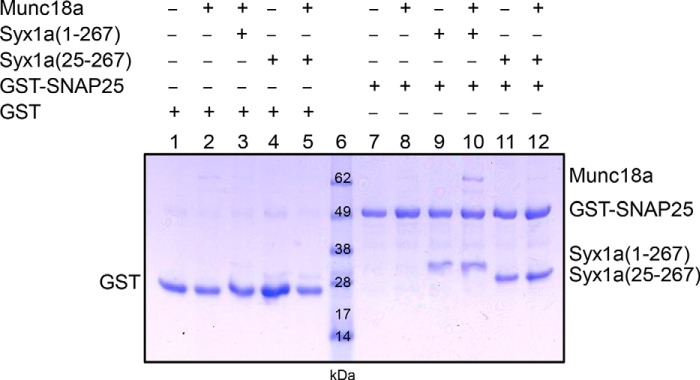
**Munc18a binds soluble t-SNAREs and requires the Stx1a(1–267) N-terminal residues.** Bead fractions (combined with SDS and boiled prior to loading) of GST (*lanes 1–5*) and GST-SNAP-25 (*lanes 7–12*) with Stx1a(1–267) (*lanes 3*, *9*, and *10*) or Stx1a(25–267) (*lanes 4*, *5*, *11*, and *12*) in the presence (*lanes 2*, *3*, *5*, *8*, *10*, and *12*) and absence (*lanes 1*, *4*, *7*, *9*, *11*) of Munc18a.

Because binding of Munc18a to the ternary complex depends upon the N-terminal residues of Stx1a, we tested whether this is also true for the binary complex. Stx1a(1–267) or the N-terminally truncated mutant Stx1a(25–267) was incubated with GST-SNAP-25 overnight, and the resulting t-SNARE complex was then mixed with Munc18a and glutathione-agarose. GST-SNAP-25 bound Munc18a in the presence of Stx1a(1–267) but not Stx1a(25–267) (Fig. 4), indicating that binding of Munc18a to the t-SNARE complex is dependent on the conserved N-terminal residues of syntaxin-1A.

**FIGURE 5.**
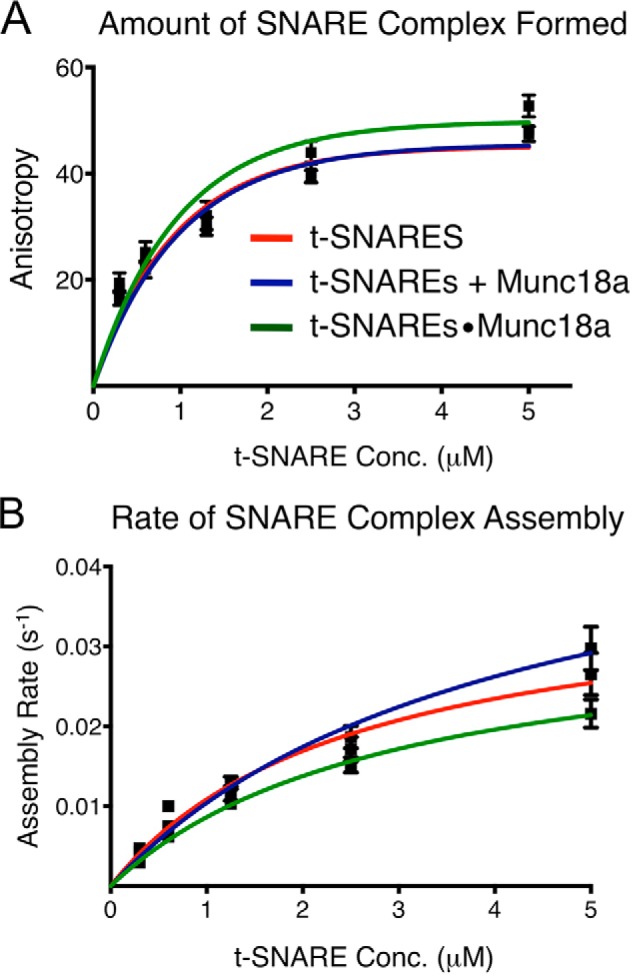
**Cytoplasmic SNARE complex formation starting from cytoplasmic, soluble t-SNAREs with and without Munc18a.** The amount of cytoplasmic SNARE complex formed (*A*) and initial rate of SNARE complex formation (*B*) were determined using fluorescence anisotropy with synaptobrevin-2(1–96)-A488. SNARE complex formation from three starting states was compared: t-SNAREs in the absence of Munc18a (*red*); t-SNAREs with sequential addition of Munc18a (*blue*); and t-SNAREs in a preformed complex with Munc18a, where Munc18a, Stx1a(1–267), and SNAP-25 were used in equal proportions (*green*). We note that protein constructs and molar ratios in this work differ from those in Ref. 70, so the rates of SNARE complex formation cannot be compared directly.

**FIGURE 6.**
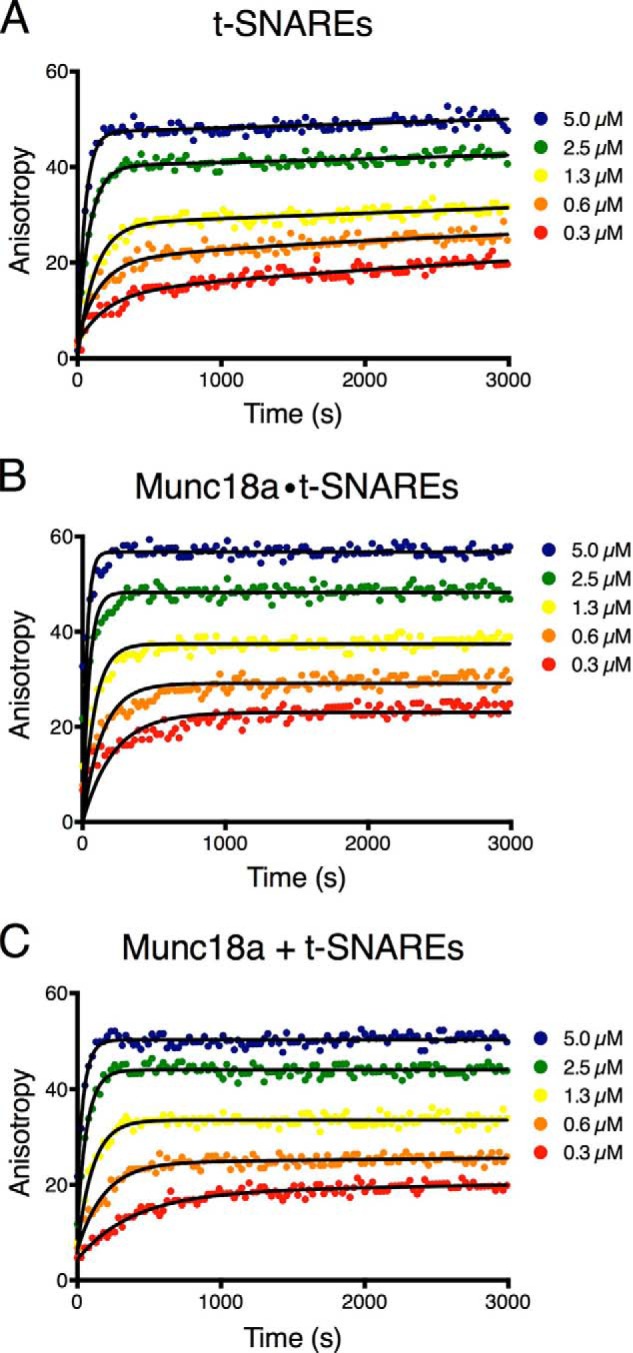
**Munc18a has no effect on the kinetics of cytoplasmic (soluble) SNARE complex formation.** Representative traces of fluorescence anisotropy experiments monitoring SNARE complex formation with synaptobrevin-2(1–96)-A488 starting from t-SNARE complex (*A*), preformed Munc18a-t-SNARE complex (*B*), and t-SNARE complex with separate addition of Munc18a (*C*). *Colored dots*, experimental data; *lines*, exponential fits of the curves.

Having established that Munc18a binds to the cytoplasmic (non-membrane anchored) t-SNARE complex, we monitored cytoplasmic ternary SNARE complex formation in the absence or presence of Munc18a. The kinetics of ternary SNARE complex formation was measured by fluorescence anisotropy, by adding fluorescently labeled synaptobrevin-2 cytoplasmic domain (synaptobrevin-2(1–96)-Alexa488) to a preformed cytoplasmic t-SNARE complex consisting of Stx1a(1–267) and SNAP-25. An increase in anisotropy, which is positively correlated with an increase of molecular mass, indicates the formation of SNARE complex. Using a 1:1 molar ratio of Stx1a(1–267) and SNAP-25 present at 6–100-fold molar excess over synaptobrevin-2(1–96)-Alexa488, we monitored the rate of ternary SNARE complex formation under three conditions (Figs. 5 and 6): 1) no Munc18a (Fig. 5, *red line*); 2) incubating Munc18a with the t-SNARE complex for 0.5–2 h prior to initiating the complex formation reaction (Fig. 5, *blue line*); and 3) starting from a gel filtration-purified 1:1:1 complex of Munc18a·Stx1a(1–267)·SNAP-25 (Fig. 5, *green line*). As a control, we confirmed that adding Munc18a to Stx1a(1–267) before adding SNAP-25, using a 1:1:1 molar ratio of Munc18a·Stx1a(1–267)·SNAP-25 over a range of concentrations, inhibits neuronal SNARE complex formation (31).

In all three cases, we found no significant difference in the amount of neuronal SNARE complex formed or the rate of complex formation (Fig. 5). In the case of sequentially added Munc18a, the protein concentrations correspond to Munc18a·t-SNARE binding occupancies of 20–80%; we were not able to achieve saturated binding due to the insolubility of Munc18a at higher concentrations. However, the results were no different starting from fully saturated complexes with a 1:1:1 molar ratio of Munc18a·Stx1a(1–267)·SNAP-25. In summary, Munc18a has no detectable effect on the rate of cytoplasmic, soluble SNARE complex formation starting from the soluble, non-membrane-anchored t-SNARE complex, and Munc18a binds to the t-SNARE complex with similar affinity as to the ternary t-SNARE complex.

##### Munc18a Reduces SV-PM Vesicle Association

**FIGURE 7.**
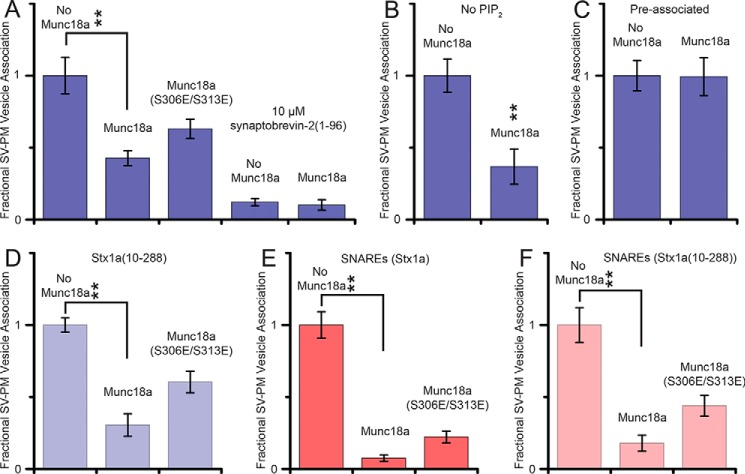
**Vesicle association efficiency between SV vesicles and immobilized PM vesicles.** *A–F*, SV vesicle association efficiency was measured by counting associated SV vesicles per field of view (45 μm × 90 μm) in the presence of 2 μm complexin. Each *panel* corresponds to measurements that were performed on the same surface and with exactly the same vesicle concentration and incubation time (*i.e.* experiments within a particular panel are comparable, whereas caution should be used when comparing different panels because they were from different protein and surface preparations). Consequently, each bar chart was normalized by the mean value of the respective condition without Munc18. For details of the incubation periods, see “Experimental Procedures.” *A*, SV vesicles associated with PM vesicles with no Munc18, with Munc18, and with Munc18a(S306E/S313E) or supplemented with 10 μm soluble synaptobrevin(1–96) in addition to the reconstituted full-length synaptobrevin-2. *B*, SV vesicles associated with PM vesicles with reconstituted Stx1a·SNAP-25 but not containing PIP_2_. *C*, SV vesicles preassociated with PM vesicles and then incubated in the flow chamber with or without Munc18a for (see “Experimental Procedures” for details). *D*, same as *A* but with PM vesicles with reconstituted Stx1a(10–288)·SNAP-25. *E*, SV vesicles with reconstituted synaptobrevin-2, but without synaptotagmin-1, associated with PM vesicles with reconstituted Stx1a·SNAP-25. *F*, same as *E* but with PM vesicles with reconstituted Stx1a(10–288)·SNAP-25. *Error bars*, S.D. values over 10–15 experiments in random locations of the same flow channel. **, *p* < 0.01 using Student's *t* test.

Although we did not observe an effect of Munc18a on the Ca^2+^-triggered fusion histograms and spontaneous fusion probabilities (Fig. 1), we found that the number of associated SV vesicles was reduced when Munc18a had been preincubated with the immobilized PM vesicles; in fact, we compensated for this reduction in associated SV vesicles by increasing the SV vesicle concentration accordingly compared with control without Munc18a (see “Experimental Procedures”). We studied this effect quantitatively by using a single vesicle-vesicle association assay that enabled higher throughput (53, 54). Similar to the fusion experiments discussed above, immobilized PM vesicles were preincubated with Munc18a before SV vesicles along with complexin-1 (see “Experimental Procedures” for details). The number of associated SV vesicles per field of view was counted and used as a measure of association efficiency between pairs of free SV and immobilized PM vesicles. For a series of conditions, the same surface preparation and immobilized PM vesicles were used, and the free SV vesicle concentrations and incubation times were kept the same as well. Because experiments with different protein and surface preparations cannot be compared directly, we plotted the results as fractional probabilities relative to the respective controls without Munc18a for each series of experiments that used the same surface and PM vesicle preparation (Fig. 7).

Munc18a reduced the number of associated SV-PM vesicles compared with the case without Munc18a (Fig. 7*A*). As a control, when the cytoplasmic domain of synaptobrevin-2 (synaptobrevin-2(1–96)) was added to the immobilized PM vesicles at 10 μm concentration, SV-PM vesicle association was essentially abolished regardless of the presence of Munc18a (Fig. 7*A*). This experiment confirms that SV-PM vesicle association depends on *trans*-SNARE complex formation because the soluble synaptobrevin-2(1–96) fragment blocks *trans*-SNARE complex formation by sequestering t-SNARE (syntaxin-1A·SNAP-25) complexes.

A larger reduction by Munc18a on vesicle association was observed when SV vesicles were reconstituted with only synaptobrevin-2 (*i.e.* without synaptotagmin-1) (Fig. 7*E*). Thus, the presence of synaptotagmin-1 masks part of the effect of Munc18a on SV-PM vesicle association. One possible explanation could be that the interaction between reconstituted synaptotagmin-1 in the SV vesicles and the membrane of the immobilized PM could be responsible. Synaptotagmin-1 interacts with membranes containing PIP_2_ even in the absence of Ca^2+^ (57, 58), so we performed an experiment without PIP_2_ in the PM vesicle preparation. However, Munc18a reduced vesicle association to a similar degree (Fig. 7*B*) as that observed in the presence of PIP_2_, implying that the effect by Munc18 on vesicle association is not caused by interference of the synaptotagmin-PIP_2_ interaction. Finally, Munc18a had no effect on SV-PM vesicle association when it was added *after* vesicles had been associated by *trans*-SNARE complex formation (Fig. 7*C*).

##### Munc18a Sequesters Syntaxin-1A from Membrane-reconstituted t-SNARE Complex

The reduction of SV-PM vesicle association by Munc18a was similar when the N-terminally truncated Stx1a(10–288) construct was used (Fig. 7, *D* and *F*). Because the observed reduction of SV-PM vesicle association does not depend on the N terminus of syntaxin-1A, yet the interaction between Munc18a and the t-SNARE complex depends on it (Fig. 4), the effect of Munc18a cannot be due to its interaction with the t-SNARE complex. Thus, we asked whether the observed reduction in SV-PM vesicle association could instead be caused by a sequestration reaction where Munc18a would capture closed syntaxin-1A and, thus, release SNAP-25.

**FIGURE 8.**
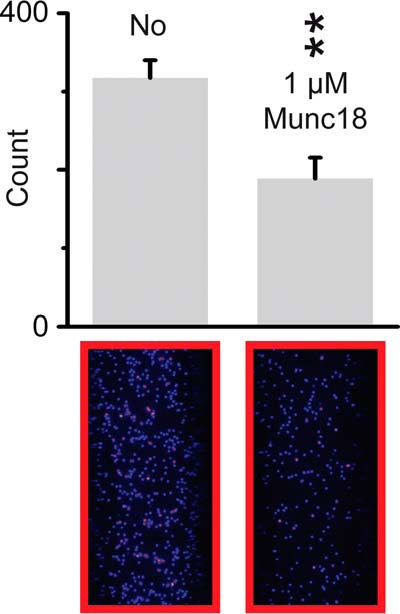
**Munc18a sequesters syntaxin-1A and releases SNAP-25 from t-SNARE complex that is reconstituted in PM vesicles.** *Top panels*, shown are the average number of labeled SNAP-25 molecules from 10 random imaging locations in the same sample channel before and after (120 min) 1 μm Munc18 addition. *Bottom panels*, representative fields of view. *Error bars*, S.D. Note that a *purple spot* indicates higher brightness. **, *p* < 0.01 using Student's *t* test.

To test this hypothesis, we labeled SNAP-25 at residue 76 and reconstituted a mixture of labeled and unlabeled SNAP-25 molecules into PM vesicles together with unlabeled syntaxin-1A. The same reconstitution and immobilization protocols were performed as for all other single vesicle experiments in this work. We adjusted the ratio of labeled to unlabeled SNAP-25 molecules in order to obtain at most one labeled SNAP-25 molecule per PM vesicle. Note that we used a soluble SNAP-25 construct (*i.e.* without palmitoylation), so once a SNAP-25 molecule was dislodged from a t-SNARE complex it was expected to diffuse away from the surface. We monitored fluorescence from single SNAP-25 molecules before and 120 min after the Munc18a addition (Fig. 8). Consistent with our hypothesis of syntaxin-1A sequestration, we observed that incubation of Munc18a results in reduction of about 50% of surface-localized SNAP-25 molecules.

##### Effects of Reducing the Munc18a-Stx1a Interaction by Phosphorylation

**FIGURE 9.**
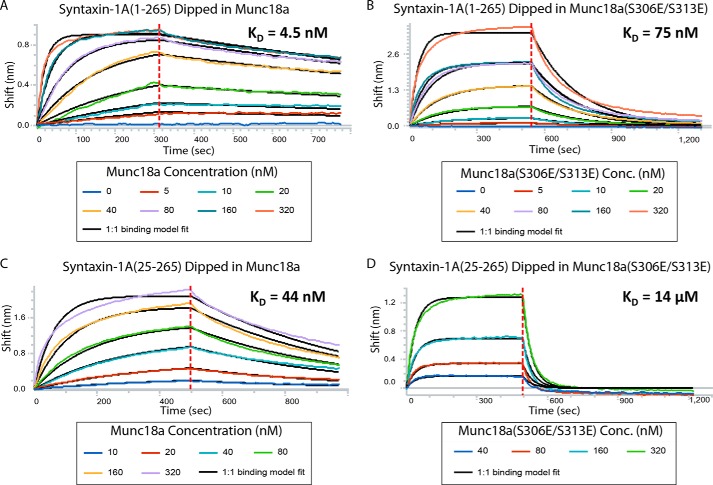
**Bio-layer interferometry experiments of Munc18a and Munc18a(S306E/S313E) binding to syntaxin-1A(1–265) or syntaxin-1A(25–265) constructs that had been surface-immobilized with a C-terminal biotin tag.** Sensograms are fit to a 1:1 binding model (*black lines*). Specified concentrations of Munc18a were used. The dissociation constant of wild type Munc18a to Stx1a(1–265) is 4.5 nm (*A*), that of Munc18a(S306E/S313E) to Stx1a(1–265) is 75 nm (*B*), that of Munc18a to Stx1a(25–265) is 44 nm (*C*), and that of Munc18a(S306E/S313E) to Stx1a(25–265) is 1,400 nm (*D*). Note that binding of Munc18a to N-terminally truncated syntaxin-1A should not be affected by the different constructs (Stx1a(25–265) *versus* Stx1a(10–288)) used in bio-layer interferometry binding experiments and in the fusion and vesicle association experiments (see the supplemental material in Ref. 31).

Phosphorylation of Munc18a greatly reduces interaction with syntaxin-1A (41), and a phosphorylation-mimicking mutant Unc-18(S322E) of *Caenorhabditis elegans* Unc-18 (S322E) reduces binding to syntaxin-1A. However, the role of phosphorylation in neurons was found to be relatively subtle; the two phosphorylation sites in Munc18a appear to be important for post-tetanic potentiation (59). In our system, similar to wild type Munc18a, the phosphorylation mimic mutant of Munc18a(S306E/S313E) had no effect on the normalized Ca^2+^-triggered fusion histograms (Fig. 1, *A–F*), the synchronization time constant (Fig. 1*G*), or the probability of spontaneous fusion (Fig. 1*H*), regardless of whether full-length Stx1a or the N-terminally truncated Stx1a(10–288) fragment were used. The phosphorylation mimic Munc18a(S306E/S313E) also reduced vesicle association but to a lesser degree (Fig. 7*A*). The lessened effect on SV-PM vesicle association by the phosphorylation mimic is correlated with its reduced affinity to syntaxin-1A compared with wild type Munc18a, as determined by bio-layer interferometry experiments (Fig. 9). Thus, these experiments further corroborate that the sequestration of syntaxin-1A by Munc18a is related to the interaction between Munc18a and syntaxin-1A.

The reduction of SV-PM vesicle association by Munc18a(S306E/S313E) was similar when the N-terminally truncated syntaxin-1A construct Stx1a(10–288) was used (Fig. 7, *D* and *F*). Moreover, the affinity of the phosphorylation mimic Munc18a(S306E/S313E) to the N-terminally truncated syntaxin-1A construct was reduced relative to wild-type syntaxin-1A (Fig. 9). This suggests that the syntaxin-1A sequestration reaction by Munc18a(S306E/S313E) is independent of the presence of the N-terminal residues of syntaxin-1A, as concluded above for wild type Munc18a.

## DISCUSSION

Our single vesicle-vesicle content-mixing assay enabled us to discriminate between effects caused by vesicle association and intrinsic fusion rates, thus overcoming a key deficiency of previous ensemble fluorescence methods, where both effects are convoluted (21). Using our single vesicle-vesicle content-mixing assay, we found that Munc18a has no effect on both spontaneous and Ca^2+^-triggered fusion rates with neuronal SNAREs, complexin-1, and synaptotagmin-1 (Fig. 1).

### 

#### 

##### Role of the Syntaxin-1A N Terminus

The N terminus of syntaxin-1A plays only a small role in the binding affinity between Munc18a and closed syntaxin-1A (30, 31). In contrast, the N terminus is essential for the interaction between Munc18a and ternary neuronal SNARE complex (31); for the interaction between Munc18a and t-SNARE complex (Fig. 4); and for co-localization of Munc18a, SNAP-25, and syntaxin-1A (32). Although the N terminus is required for recruitment of Munc18, its function could be physically separated in neuromuscular junctions of *C. elegans* by using “split” syntaxin transgenes (18). Moreover, point mutations of Munc18a that interfere with binding to the syntaxin-1A N terminus and disrupt binding to the assembled SNARE complexes all support normal docking, priming, and fusion of synaptic vesicles as well as normal synaptic plasticity in rescue experiments with neuronal cultures using autaptic munc18a null mutant neurons (60). Consistent with these previous results, in our experiments, the syntaxin-1A N terminus had no effect on fusion rates (Fig. 1) and vesicle association (Fig. 7).

##### Munc18a Sequesters Syntaxin-1A from Reconstituted t-SNARE Complex

When we added Munc18a to immobilized PM vesicles with reconstituted syntaxin-1A and SNAP-25, we observed reduction of SV-PM vesicle association on a ∼30-min time scale (Fig. 7). This result suggested that Munc18a reduces vesicle association by sequestration of syntaxin-1A (*i.e.* releasing SNAP-25 from t-SNARE complex). We independently confirmed the sequestration of syntaxin-1A by Munc18a by a single molecule experiment (Fig. 8). Moreover, the phosphorylation mimic mutant of Munc18a with reduced affinity to syntaxin-1A resulted in less reduction of vesicle association (Fig. 7*A*), corroborating the notion of syntaxin-1A sequestration.

The interaction between Munc18a and syntaxin-1A lacking the N-peptide is strong (*K_d_* = 10 nm) albeit reduced compared with full-length syntaxin-1A (*K_d_* = 1.4 nm) (31). Moreover, the crystal structure and solution scattering analysis of the complex between syntaxin-1A and Munc18a is similar in the presence or absence of the syntaxin N-terminal residues (56). In both cases, Munc18a is expected to sequester syntaxin-1A and interact with it in a closed conformation, consistent with our results.

Sequestration of the cytoplasmic, soluble fragment of syntaxin-1A from t-SNARE complex by Munc18a was previously observed by nuclear magnetic resonance spectroscopy on a 11-h time scale (Fig. 1 in Ref. 8) and by a FRET-based assay on a ∼30-min time scale using separate SNAP-25 N- and C-terminal SNARE helices (Fig. S1*B* in Ref. 8). Thus, there is considerable variation of the time scale of syntaxin-1A sequestration depending on the constructs and experimental conditions; this probably explains why there is no apparent sequestration of syntaxin-1A (which would produce much larger enthalpy changes and a smaller *K_d_*) (56) on the ∼1-h time scale of our ITC experiment (Fig. 3).

##### A Possible Molecular Mechanism for the Sequestration of Syntaxin-1A by Munc18a

Previously, the conformations of 1:1 binary (syntaxin-1A·SNAP-25) complex were studied by single molecule FRET experiments using binary complexes that were reconstituted into supported bilayers (61). These experiments revealed three configurations of the 1:1 syntaxin-1A·SNAP-25 binary complex; one configuration corresponds to a parallel three-helix bundle, whereas the other two are flexible configurations with one of the SNAP-25 SNARE domains dissociated. Occasional transitions between these configurations were observed. Adding synaptobrevin-2 entirely suppressed the flexible configurations. The addition of complexin-1, the MUN domain of Munc13, Munc18a, or synaptotagmin-1, also reduced the flexible configurations to varying degrees. The single molecule experiments with labeled SNAP-25 presented here (Fig. 8) suggest that Munc18a is capable of sequestering a fraction of syntaxin-1A molecules because we observed a loss of surface-localized SNAP-25 upon incubation with Munc18a. Despite the prolonged incubation with Munc18a, this sequestration of syntaxin-1A molecules was only partial. Combined with the results presented in Ref. 61, we suggest that sequestration of syntaxin-1A by Munc18a occurs only for the fraction of 1:1 syntaxin-1A·SNAP-25 complexes that are in the flexible configurations during the incubation period; presumably, the kinetic barrier to sequester syntaxin-1A by Munc18a is lower for the flexible configurations.

##### Is There an Interaction between Munc18a and Synaptobrevin-2?

Previously, it had been suggested that Munc18a interacts with synaptobrevin-2 based on ensemble lipid-mixing assays (17) and line broadening in solution NMR experiments (62). The lack of a significant difference in affinity between binary and ternary neuronal SNARE complex (Fig. 3) or an effect on fusion rates (Fig. 1) is consistent with the very weak (*K_D_* ∼15 μm) affinity reported for the Munc18a-synaptobrevin interaction (62). Because our experiments were performed well below the reported *K_D_* of 15 μm for this interaction, if the intracellular Munc18a concentration were substantially higher, it is possible that contacts between Munc18a and synaptobrevin-2 could have some role in fusion.

##### Effect of Munc18 on Vesicle Association Is Independent of PIP_2_

PIP_2_ is a charged lipid that is enriched in the plasma membrane and may promote synaptic vesicle recruitment to the active zone (63). When PIP_2_ was excluded from PM vesicles in our vesicle association assay, the overall association efficiency was greatly reduced, such that a much higher SV vesicle concentration had to be used in order to obtain a significant number of associated SV-PM vesicle pairs. However, the ratio of vesicle association with and without Munc18a was unchanged (Fig. 7, compare *A* and *B*), illustrating that the effect of Munc18a on vesicle association is PIP_2_-independent. Synaptotagmin-1 interacts with PIP_2_-containing membranes even in the absence of Ca^2+^ (57) and is thus expected to increase association between SV and PM vesicles, as we observed. When synaptotagmin-1 was eliminated from SV vesicles, the same fractional vesicle associations were observed for control, Munc18a, and Munc18a(S306E/S313E) (Fig. 7, compare *A* and *E*), consistent with the notion that the effect of Munc18a in these experiments is to sequester syntaxin-1A and release SNAP-25. However, in the absence of synaptotagmin-1, the reduction of SV-PM vesicle association by Munc18a was more pronounced, suggesting that synaptotagmin-1 interactions with t-SNARE (syntaxin-1A·SNAP-25) complexes (64) can compensate the partial reduction by Munc18a; as mentioned above, this interaction also stabilizes t-SNARE complexes (65).

##### Munc18a Has No Effect on Spontaneous and Ca^2+^-triggered Fusion

Recent work by Lou *et al.* (71) shows that Munc18a stimulates single vesicle-vesicle lipid mixing with neuronal SNAREs in the absence of synaptotagmin-1 but that there is no stimulatory effect on single vesicle lipid mixing by Munc18a in the presence of neuronal SNAREs and synaptotagmin-1. Moreover, their experiments with SNAREs that were reconstituted in nanodiscs offer an explanation for the enhancement of lipid mixing: Munc18a increases a state of the *trans*-SNARE complex where the C-terminal ends of the SNARE domains are close to each other in the absence of synaptotagmin-1, whereas in the presence of synaptotagmin-1, Munc18 has no effect on the states of the *trans*-SNARE complex (see Fig. 1, *C* and *D*, in Ref. 71). Their results suggest that the observed increase in lipid mixing is a consequence of increased vesicle association that occurs when Munc18a increases the C-terminal zippering probability of the neuronal *trans*-SNARE complex. Although we did not see an effect on soluble ternary SNARE complex formation by Munc18 (Figs. 5 and 6), we can reconcile these observations by noting that assembly of the neuronal SNARE complex is paused under loaded conditions (*i.e.* when the repulsive forces between solvated bilayers oppose *trans*-SNARE complex formation) (66, 67). Thus, the C-terminal zippering probability is affected by the presence of the reconstituted transmembrane domains, and in the absence of the transmembrane domains, one would not expect to see the intermediate state observed by Lou *et al.* (71), consistent with our results.

Parisotto *et al.* (20) showed that spontaneous ensemble lipid mixing (*i.e.* at zero Ca^2+^) is increased in the absence of complexin-1, whereas this effect is smaller in the presence of complexin-1 (see Fig. 3, *A* and *B*, in Ref. 20). However, the subsequent further increase in ensemble lipid mixing upon Ca^2+^ injection is similar, regardless of the presence of Munc18a. Moreover, Fig. 3*C* in Ref. 20 shows that the subsequent increase in ensemble content mixing upon Ca^2+^ injection is also unaffected by the presence or absence of Munc18a. Thus, the lack of an effect on Ca^2+^ triggered ensemble content mixing is entirely in agreement with our single vesicle-vesicle Ca^2+^-triggered fusion results (Fig. 1, *A–C* and *G*) that show no increase in synchronization of Ca^2+^-triggered fusion with neuronal SNAREs, synaptotagmin-1, and complexin-1 upon the addition of Munc18a. Moreover, Figs. 3 and 4 in Ref. 71 show little effect of Munc18a on single vesicle-vesicle lipid and content mixing for spontaneous fusion in the presence of neuronal SNAREs and synaptotagmin-1 (normalized by the number of associated/docked vesicles).

We emphasize that the data of Parisotto *et al.* (20) showing an increase in ensemble lipid mixing by Munc18a was not normalized by the number of associated/docked vesicles, so it can be explained by an increase in vesicle association (*i.e.* more vesicles are associated, resulting in a larger signal) rather than an effect on hemifusion or fusion *per se*. This explanation also applies to other previously published results (16, 68). Moreover, we speculate that the apparent difference in the degree of ensemble lipid mixing can be explained by the competition of the two processes, the enhancement of neuronal SNARE zippering in the absence of synaptotagmin-1 *versus* the sequestration of syntaxin-1A by Munc18a; different experimental conditions may shift the balance between these two processes. For example, the requirement of preincubation of vesicles at 4 °C for the ensemble lipid mixing experiments by Shen *et al.* (17) could arise from shifting the balance between Munc18a-enhanced SNARE zippering *versus* Munc18a-induced sequestration of syntaxin-1A.

NSF and αSNAP will disassemble the t-SNARE complex (8); other experiments suggest that Munc18a then prevents further action of NSF/αSNAP by sequestering syntaxin-1A and locking it into the closed confirmation and preventing *trans*-SNARE formation until synaptobrevin-2 and Munc13 arrive (8). We note that the amount of ensemble lipid mixing was roughly the same for a minimal system consisting only of neuronal SNAREs, the C2AB fragment of synaptotagmin-1, and 0.5 μm Ca^2+^ and for the more complete system consisting of the same proteins plus NSF, αSNAP, Munc18a, and Munc13 (see Fig. 4*D* in Ref. 8). One of the reasons for our study was to investigate whether the ensemble lipid-mixing method may have masked an effect of Munc18a on content mixing. Our results now conclusively show that Munc18a has no effect on intrinsic spontaneous and Ca^2+^-triggered fusion rates in conjunction with neuronal SNAREs, synaptotagmin-1, and complexin-1. Our study was enabled by our single vesicle-vesicle fusion system, where effects on vesicle association can be separated from effects on spontaneous and Ca^2+^-triggered fusion (40, 45, 51).

Südhof (69) noted in his 2013 Nobel Lecture, “The fact that SM proteins are required continuously during SNARE-complex assembly argues for a role either in organizing proper SNARE-complex assembly and in preventing dead-end inappropriate SNARE complexes, or in catalyzing lipid mixing during fusion. At present, no conclusive data argue one way or the other, and this question will clearly keep many of us busy for years to come.” Because our results show that Munc18a has no effect on fusion in conjunction with neuronal SNAREs, synaptotagmin-1, and complexin-1, the following consensus view emerges. Munc18a primarily plays a role in proofreading of *trans*-SNARE complexes rather than directly in fusion itself. This notion has also been suggested for the SM protein-containing HOPS complex in the context of vacuolar fusion (27). However, future reconstitution experiments with all factors involved in synaptic vesicle fusion will have to be carried out in order to study the proposed proofreading role of Munc18a.

## Footnotes

7 The abbreviations used are:

NSF: *N*-ethylmaleimide-sensitive factor
Syt1: synaptotagmin-1
SM: Sec1/Munc18
αSNAP: soluble NSF adaptor protein
IPTG: isopropyl β-d-1-thiogalactopyranoside
Ni-NTA: nickel-nitrilotriacetic acid
OG: *n*-octyl-β-d-glucopyranoside
TEV: tobacco etch virus
TCEP: tris(2-carboxyethyl)phosphine
ITC: isothermal titration calorimetry
SV: synaptic vesicle-mimicking
PM: plasma membrane-mimicking
PIP_2_: phosphatidylinositol 4,5-bisphosphate.
